# Functional Validation of a *CC2D2A* Non-Canonical Splice-Site Variant Supports Prenatal Molecular Testing in Joubert Syndrome

**DOI:** 10.3390/diagnostics16193150

**Published:** 2026-09-28

**Authors:** Fang Wang, Jiaying Zhang, Chao Lu, Xinlian Wang, Huimin Gao, Xinyu Fu, Yan Liu, Meng Zhang, Yujiao Chen, Lili Li, Bo Cao, Minna Luo, Yousheng Yan

**Affiliations:** 1 Department of Medical Genetics, Beijing Obstetrics and Gynecology Hospital, Capital Medical University, Beijing Maternal and Child Health Care Hospital, Beijing 100026, China; wangfang1987@mail.ccmu.edu.cn (F.W.); zhangjiaying800@mail.ccmu.edu.cn (J.Z.); ghm@mail.ccmu.edu.cn (H.G.); f15103702218@mail.ccmu.edu.cn (X.F.); zhmengcq@ccmu.edu.cn (M.Z.); chenyujiao0924@mail.ccmu.edu.cn (Y.C.); 18706380553@163.com (L.L.); 13264340778@163.com (B.C.); 2 National Human Genetic Resources Center, National Research Institute for Family Planning, Beijing 100089, China; luchao0107@sina.com; 3 Department of Radiology, Beijing Obstetrics and Gynecology Hospital, Capital Medical University, Beijing Maternal and Child Health Care Hospital, Beijing 100026, China; wangxinlian@ccmu.edu.cn; 4 Department of Prenatal Diagnosis, Beijing Obstetrics and Gynecology Hospital, Capital Medical University, Beijing Maternal and Child Health Care Hospital, Beijing 100026, China; liuyan1292@ccmu.edu.cn

**Keywords:** Joubert syndrome, *CC2D2A*, splicing variant, minigene assays, prenatal diagnosis

## Abstract

**Background:** Joubert syndrome (JS) is a genetically heterogeneous ciliopathy wherein non-canonical splice-site (NCSS) variants are frequently reported as variants of uncertain significance (VUS), complicating prenatal counseling. We aimed to clarify the clinical significance of an NCSS in *CC2D2A* through functional validation. **Methods:** Trio-WES was performed on a 13-year-old female proband with clinically diagnosed JS. Candidate variants were validated by Sanger sequencing and familial segregation. An in vitro minigene assay and RT-PCR on patient peripheral blood RNA were used to assess the splicing effect of c.439-5T>G. Variant classification followed the ACMG/AMP guidelines using the ClinGen quantitative Bayesian framework. **Results:** Compound heterozygous *CC2D2A* variants were identified: c.439-5T>G (maternal) and c.4732G>A (p.(Val1578Met), paternal); both were initially classified as VUS. Minigene analysis showed that c.439-5T>G caused aberrant splicing, retaining the intronic nucleotides c.439-4_439-1 (ATAG) at the exon 6–7 junction (r.438_439insauag) and introducing a frameshift and premature termination (p.(Pro147IlefsTer14)). Patient RNA confirmed this pattern. c.439-5T>G was reclassified as likely pathogenic (PVS1_VeryStrong + PM2_Supporting). c.4732G>A remained a VUS (PM3 + PM2_Supporting + PP3) on an independent allele basis; its trans configuration with the likely pathogenic allele supported a family-specific biallelic explanation but did not establish the pathogenicity of the missense allele. The fetus in a subsequent pregnancy was confirmed to carry both variants, establishing the compound heterozygous state. **Conclusions:** Minigene-based validation provided evidence to reclassify a *CC2D2A* NCSS from VUS to likely pathogenic, enabling variant-directed prenatal testing and informed reproductive counseling in this JS family. The paternal missense variant c.4732G>A (p.(Val1578Met)) remains a VUS; its trans configuration with the likely pathogenic allele supports, but does not establish, a biallelic explanation. This study illustrates how functional assays can clarify splicing variants that in silico predictions alone cannot reliably interpret.

## 1. Introduction

Joubert syndrome (JS; OMIM #213300) is a rare autosomal recessive neurodevelopmental ciliopathy characterized by the pathognomonic “molar tooth sign” on axial brain MRI, accompanied by hypotonia, ataxia, developmental delay, and oculomotor apraxia [1,2]. Over 40 genes have been associated with JS, reflecting its marked genetic heterogeneity [3,4]. Among the major genes, *CC2D2A* (Coiled-Coil and C2-Domain-Containing 2A) accounts for a notable proportion of genetically confirmed cases [5,6]. The *CC2D2A* protein resides at the transition zone of the primary cilium, and biallelic pathogenic variants disrupt ciliary trafficking and Hedgehog signaling [7]. At the group level, cohort data suggest that severe renal, retinal, and hepatic involvement is less frequent in *CC2D2A*-related JS than in some other JS subtypes; however, developmental delay is nearly constant, neurodevelopmental outcomes vary widely even within *CC2D2A*-related JS, and individual prognosis cannot be reliably predicted [5]. Molecular characterization therefore remains important for informed prenatal counseling in these families.

High-throughput sequencing, particularly trio whole-exome sequencing (Trio-WES), has improved the molecular diagnostic yield for JS to approximately 60% to 90% [3,6,8] and is increasingly used in selected pregnancies with fetal structural anomalies [9,10]. However, many patients remain without a molecular diagnosis. Some unresolved cases harbor cryptic variants, including intragenic copy number variants and deep intronic splice-site variants, that are missed by standard ES analysis pipelines [6]. In other cases, NCSS located beyond the canonical ±1/±2 dinucleotides are detected but classified as VUS, making them difficult to interpret in clinical practice [11,12,13]. Bioinformatic predictions alone are often insufficient to establish the pathogenicity of NCSS variants, as these may disrupt splicing regulatory elements without altering consensus sequences [14,15]. Targeted reanalysis of ES data can identify cryptic variants missed by initial analysis and has increased diagnostic yield in both postnatal and prenatal unresolved cohorts [6,16]. For NCSS variants already classified as VUS, functional assays, particularly minigene splicing experiments, provide direct evidence of aberrant splicing; under the ClinGen SVI Splicing Subgroup recommendations, such RNA-level splicing evidence is captured through the PVS1 criterion, whereas PS3 applies to independent downstream functional assays, facilitating variant reclassification [17,18,19,20,21].

In this study, we identified compound heterozygous *CC2D2A* variants (c.439-5T>G and c.4732G>A/p.(Val1578Met)) in a Chinese proband with JS. The intronic variant c.439-5T>G, located five nucleotides upstream of the exon 7 acceptor site, was classified as a VUS. We combined bioinformatic prediction, a minigene splicing assay, and RT-PCR on patient peripheral blood RNA to determine whether this NCSS affects splicing. We aimed to provide functional evidence to support reclassification of this variant and to evaluate its implications for prenatal counseling in this family.

## 2. Methods

### 2.1. Ethics Statement

This study was approved by the Medical Ethics Committee of Beijing Obstetrics and Gynecology Hospital, Capital Medical University (No. 2023-KY-009-01). Written informed consent was obtained from the parents of the proband for clinical genetic testing. Residual biological samples from routine clinical testing and de-identified clinical data were used for research under the approved protocol. The study adhered to the Declaration of Helsinki.

### 2.2. Patients and Sample Collection

The 13-year-old female proband was diagnosed with JS at the Department of Medical Genetics, Beijing Obstetrics and Gynecology Hospital, based on characteristic clinical manifestations and typical MRI findings. Peripheral blood (2 mL) was collected from the proband and her parents in EDTA tubes. Amniotic fluid (10 mL) was collected by amniocentesis at 18 weeks of gestation in the subsequent pregnancy for variant-directed testing of the two familial *CC2D2A* variants.

### 2.3. Trio Whole-Exome Sequencing (Trio-WES) and Bioinformatic Analysis

Genomic DNA was extracted from peripheral blood samples obtained from the proband and her parents using the Nucleic Acid Extraction and Purification Kit (AmCare Genomics Laboratory, Guangzhou, China) according to the manufacturer’s protocol. Following extraction, genomic DNA was fragmented via enzymatic digestion, and libraries were constructed using the Gene Sequencing Library Kit (AmCare, Guangzhou, China). Trio-WES was conducted on the AmCareSeq-2000 sequencing platform (AmCare, Guangzhou, China). Exonic regions were enriched using NEXome XP Panel v. 1.0 (AmCare, Guangzhou, China). Paired-end sequencing (150 bp) was performed with an average sequencing depth of 100×, with >98% of target regions covered by at least 20 reads.

Raw reads were processed with fastp software (v. 0.20.2) to trim adapters and low-quality bases (quality threshold < Q20). Clean reads were aligned to the GRCh37/hg19 reference genome using Burrows-Wheeler Aligner (BWA-MEM, v. 0.7.15). Variant calling for single nucleotide variants (SNVs) and small insertions/deletions (indels) was performed using the HaplotypeCaller module of the Genome Analysis Toolkit (GATK, v4.3.0.0). Variants with variant allele frequency (VAF) < 15% were excluded.

Variant annotation was performed using ANNOVAR (https://annovar.openbioinformatics.org/en/latest/, accessed on 15 March 2023), and filtering was performed using an in-house bioinformatics pipeline (AmCare). The clinical significance of variants was assessed against public databases including gnomAD (https://gnomad.broadinstitute.org/, accessed on 15 March 2023), ClinVar (https://www.ncbi.nlm.nih.gov/clinvar/, accessed on 15 March 2023), OMIM (https://www.omim.org/, accessed on 15 March 2023), and an internal database of 100,000 Han Chinese individuals (AmCare). Suspected mutations were validated by Sanger sequencing of PCR products on an ABI 3730xl Genetic Analyzer (Applied Biosystems, Foster City, CA, USA). The conservation and pathogenicity of the variants were predicted using SIFT, PolyPhen-2, MutationTaster, and FATHMM scores retrieved from the dbNSFP database. Splicing predictions for variants at or near splice junctions were evaluated using SpliceAI, MaxEntScan (both incorporated in dbNSFP), and Human Splicing Finder (v. 3.1). Variant classification was performed according to the ACMG/AMP guidelines (2015), with evidence strength calibrated using the quantitative Bayesian framework endorsed by the ClinGen Sequence Variant Interpretation Working Group [18,20].

### 2.4. Sanger Sequencing Validation and Familial Segregation Analysis

For independent validation of candidate variants and familial segregation analysis, genomic DNA was extracted from peripheral blood samples of the proband and her parents using the Nucleic Acid Extraction and Purification Kit (Cat. No. CWY005S, Jiangsu Cowin Biotech, Taizhou, China). Fetal genomic DNA was extracted from amniotic fluid cells using the QIAamp DNA Mini Kit (Cat. No. 51304, QIAGEN, Hilden, Germany). Target fragments were amplified by PCR, and the PCR products were analyzed via 2% agarose gel electrophoresis. Sanger sequencing was performed for the c.439-5T>G and c.4732G>A (p.(Val1578Met)) sites using the primers listed in Table 1. Sequence data were analyzed with Chromas software (v. 2.6.6.0) and aligned to the *CC2D2A* reference sequence (NM_001378615.1, MANE Select).

### 2.5. Minigene Splicing Assay

To investigate the potential splicing effect of the intronic variant c.439-5T>G, we constructed minigene plasmids from genomic DNA of the proband and a healthy control. Two target gene fragments, *CC2D2A*-A and *CC2D2A*-B, were amplified using two specific primer sets (Table 1). The minigene regions spanning *CC2D2A* exons 6–8 and flanking introns were cloned into the pMini-CopGFP vector (Beijing Hitrobio Biotechnology, Beijing, China) using the ClonExpress II One Step Cloning Kit (Cat. No. C112-02, Vazyme, Nanjing, China). The vector had been linearized by double digestion with BamHI and XhoI restriction enzymes. The resulting wild-type (WT) and mutant (MT) plasmids were verified by full-length Sanger sequencing of the complete inserts (2465 bp); the two inserts differed solely at c.439-5T>G. The construction process is illustrated in Figure 1.

Human embryonic kidney 293T (HEK293T) cells (Beijing Hitrobio Biotechnology, Beijing, China) were maintained in Dulbecco’s Modified Eagle’s Medium (DMEM, Cat. No. C12430500BT, Thermo Fisher Scientific (China) Co., Ltd., Shanghai, China) supplemented with 10% fetal bovine serum (Cat. No. ST30-3302, PAN-Biotech GmbH, Aidenbach, Germany) at 37 °C in a humidified 5% CO_2_ incubator. The minigene recombinant plasmid was mixed with Lipofectamine™ 2000 diluent (Cat. No. 11668019, Invitrogen, Thermo Fisher Scientific, Carlsbad, CA, USA) to form transfection complexes, which were then transfected into HEK293T cells. After 48 h of transfection, cells were harvested, and total RNA was extracted using the TRIzol method. The transfection experiment was performed once on pooled cells, without single-cell cloning; the qualitative splicing readout was subsequently confirmed in patient peripheral blood RNA.

### 2.6. Reverse Transcription-PCR (RT-PCR) and Sanger Sequencing of Minigene and Patient RNA

Total RNA (1 µg) from transfected HEK293T cells was reverse-transcribed into cDNA using HiScript II 1st Strand cDNA Synthesis Kit (Cat. No. R212-01, Vazyme, Nanjing, China) with oligo(dT) primers. RT-PCR of minigene transcripts was performed using the vector-specific primer MiniRT-F and *CC2D2A*-RT-R (exon 8) (Table 1). Amplification conditions: initial denaturation at 95 °C for 2 min; 30 cycles of 95 °C for 20 s, 60 °C for 30 s, and 72 °C for 60 s. Products were separated on 2% agarose gels and purified for Sanger sequencing.

For patient RNA validation, peripheral blood was collected in Tempus Blood RNA Tubes (Cat. No. 4342792, Applied Biosystems, Waltham, MA, USA), and total RNA was extracted using the Tempus Spin RNA Isolation Kit (Cat. No. 4380204, Applied Biosystems, Waltham, MA, USA) according to the manufacturer’s instructions; this workflow includes no DNase digestion step. No-RT controls were not performed; however, the forward primer spans the exon 5–6 junction and therefore has no contiguous binding site in genomic DNA, excluding amplification of any residual genomic DNA. No-template controls were included in all runs and were negative. cDNA synthesis was performed as described above. RT-PCR of endogenous *CC2D2A* transcripts used the gene-specific primers *CC2D2A*-cDNA-F, spanning the exon 5–6 junction (c.330–c.349), and *CC2D2A*-cDNA-R, annealing to exon 9 (c.748–c.767) of NM_001378615.1 (Table 1), yielding an expected wild-type product of 438 bp and a 442 bp product for transcripts carrying the 4 bp retention. Touchdown PCR was performed as follows: 94 °C for 5 min; 25 cycles of 94 °C for 30 s, 65→60 °C for 30 s (touchdown, −0.5 °C per cycle), and 72 °C for 30 s; 10 cycles of 94 °C for 30 s, 60 °C for 30 s, and 72 °C for 30 s; final extension at 72 °C for 5 min. Products were analyzed by agarose gel electrophoresis and confirmed by TA cloning followed by Sanger sequencing.

## 3. Results

### 3.1. Clinical Presentation

The proband, a female, was born in February 2012. No abnormalities were observed at birth, but psychomotor developmental delay was first noted at 5 months of age. Brain MRI at age 3 years showed the characteristic molar tooth sign (Figure 2A). Clinical findings included mild intellectual disability, strabismus, nystagmus, hypotonia, ataxia, and motor delay; no seizures and no infantile episodes of tachypnea or apnea were reported, and growth was within the normal range. Ophthalmologic examination, including funduscopy, was unremarkable; urinalysis was normal, and abdominal ultrasonography showed no hepatomegaly or hepatic fibrosis; electroretinography, renal ultrasonography, and serum renal and liver function tests were not performed. JS was diagnosed on the basis of these features and the MRI findings. At 13 years, she attended a regular school with learning support.

The molecular workup was completed before the subsequent pregnancy, and the prenatal testing described below proceeded prospectively on a variant-directed basis. The proband’s two *CC2D2A* variants were identified by trio-WES in March 2023, and the minigene assay together with patient RNA analysis was completed in October 2023, leading to reclassification of c.439-5T>G as likely pathogenic. When the mother became pregnant again in 2025, prenatal counseling was based on a likely pathogenic maternal allele and a paternal missense variant that remained a VUS. The parents were counseled that detection of both familial variants in the fetus would not, by itself, establish the diagnosis because c.4732G>A remained a VUS; accordingly, variant-directed testing was interpreted together with fetal MRI assessment. Amniocentesis at 18 weeks of gestation showed that the fetus carried both variants in trans. Fetal brain MRI at 26 weeks of gestation (SSFSE) showed a small cerebellar vermis on the sagittal image and thickened, elongated, horizontally oriented bilateral superior cerebellar peduncles on the axial image, together with an abnormally configured fourth ventricle, consistent with a prenatal molar tooth sign (Figure 2B). The family pedigree indicated autosomal recessive inheritance (Figure 2C).

### 3.2. Genetic Findings and Initial Variant Classification

Trio-WES analysis identified two heterozygous variants in the *CC2D2A* gene in the proband: c.439-5T>G and c.4732G>A (p.(Val1578Met)). Sanger sequencing confirmed both variants and demonstrated biparental inheritance in trans: c.439-5T>G was inherited from the mother, and c.4732G>A was inherited from the father (Figure 3). Both variants were also present in amniotic fluid cells from the subsequent pregnancy (Figure 3).

The c.439-5T>G variant was absent from the ESP6500, ExAC, and gnomAD databases (PM2_Supporting). Splice prediction algorithms (SpliceAI, MaxEntScan) predicted a splicing effect, but the prediction alone was not sufficiently definitive for classification (PP3). Under ACMG guidelines, these criteria led to an initial VUS classification. The c.4732G>A (p.(Val1578Met)) variant was also absent from population databases (PM2_Supporting), occurred at a conserved residue within a functional domain, and was predicted to be deleterious by multiple algorithms (PP3). It was also initially classified as a VUS.

### 3.3. Functional Validation of c.439-5T>G by Minigene Assay

To assess whether c.439-5T>G affects pre-mRNA splicing, we performed a minigene assay. HEK293T cells were transfected with wild-type (pMini-CopGFP-WT) or mutant (pMini-CopGFP-MT) minigene plasmids. RT-PCR of RNA from transfected cells yielded a single specific product for each construct (Figure 4A). Because a 4 bp difference cannot be resolved on a 2% agarose gel, the products were identified by Sanger sequencing rather than gel mobility (Figure 4A).

Sanger sequencing of the RT-PCR products confirmed that the WT minigene yielded correctly spliced mRNA containing intact exons 6–8. In the MT minigene, aberrant splicing resulted in the retention of a 4 bp sequence (ATAG) from the 3′ end of intron 6, creating a chimeric exon6-ATAG-exon7 junction (Figure 4B,C). This corresponds to the mRNA alteration NM_001378615.1:r.438_439insauag. Inspection of the intron 6 acceptor region supports this mechanism: c.439-6 is an adenine, so the c.439-5T>G substitution creates a new AG dinucleotide at positions −6/−5; use of this cryptic acceptor extends exon 7 at its 5′ end by exactly the retained tetranucleotide (c.439-4_439-1, ATAG). This insertion produces a frameshift starting at codon 147 (p.(Pro147IlefsTer14)); the premature termination codon lies in exon 7 of 37 and is predicted to trigger nonsense-mediated mRNA decay (NMD), and any transcript escaping NMD would yield a truncated protein (Figure 4C).

### 3.4. Patient RNA Validation

To confirm that the aberrant splicing observed in the minigene system occurs in vivo, we performed RT-PCR on peripheral blood RNA from the proband, her parents, and a healthy control. On direct Sanger sequencing of the RT-PCR products, the father and the healthy control showed clean exon 6–7 junction sequences, whereas the proband and her mother, both heterozygous carriers, showed overlapping peaks immediately downstream of the junction, consistent with a mixture of normal and aberrant transcripts (Figure 4D). TA cloning of the proband’s RT-PCR products separated the two alleles: wild-type clones showed normal exon 6–7 splicing, and mutant clones showed retention of the intronic nucleotides c.439-4_439-1 (ATAG) (Figure 4E), thereby validating the minigene findings in a biological context.

### 3.5. Variant Reclassification

Minigene and patient RNA analysis showed that c.439-5T>G causes aberrant splicing, producing a frameshift and premature termination (p.(Pro147IlefsTer14)). Under the ACMG/AMP guidelines, this variant was classified as likely pathogenic using the ClinGen quantitative scoring framework: PVS1_VeryStrong (8 points) was applied according to the ClinGen SVI splicing recommendations, under which RNA-level evidence is captured through the PVS1 decision tree [21]. The Very Strong level was retained for two reasons: the PTC introduced by the 4 bp retention (p.(Pro147IlefsTer14)) lies in exon 7 of 37 on NM_001378615.1 and is predicted to trigger NMD, a position-based assignment that does not require experimental demonstration of decay [22], so the reduced-strength branches for NMD-escaping truncations do not apply; and no residual normal splicing was detected from the mutant minigene construct, in which the variant allele is expressed in isolation—all sequenced RT-PCR products carried the 4 bp retention, with no normally spliced product observed within the detection limit of the assay. Because c.439-5T>G is intronic and absent from the mature transcript, the allele-of-origin of normal transcripts cannot be determined in endogenous RNA from heterozygous carriers; completeness of the splicing defect can therefore only be assessed in a system expressing the variant allele alone, such as the minigene assay. Under the SVI recommendations, strength is reduced for a demonstrated near-complete impact, and PVS1 is withheld for an incomplete impact [21]; as no such leakiness was demonstrated, the Very Strong level was retained. PM2_Supporting (1 point) reflected its absence from population controls. The combined score of 9 points reached the likely pathogenic threshold (Table 2). The in silico splice predictions (PP3) considered at the initial assessment were not carried into the final classification, as they are not independent of the experimentally demonstrated splicing defect captured by PVS1.

Independent assessment of c.4732G>A yielded a score of 4 points: PM3 (2 points) for its trans configuration with the likely pathogenic c.439-5T>G variant in the affected proband, with phase verified by parental testing; PM2_Supporting (1 point) for absence from population databases; and PP3 (1 point) for computational evidence of a deleterious effect. PM3 was applied only once; the fetus was considered within-family segregation and was not counted as an additional PM3 observation. A score of 4 points falls within the VUS range (0–5 points) under the ClinGen framework (Table 3), reflecting the limited evidence for the missense allele in isolation. In this family, the trans configuration with the likely pathogenic c.439-5T>G variant, together with concordant prenatal imaging findings, informed prenatal counseling but did not reclassify the missense allele.

## 4. Discussion

We identified two *CC2D2A* variants in trans in a Chinese family with JS. Functional studies showed that c.439-5T>G causes aberrant splicing, supporting its reclassification as likely pathogenic, whereas c.4732G>A remained a VUS. These findings informed variant-directed prenatal testing and counseling in the subsequent pregnancy.

### 4.1. Novelty and Expansion of the CC2D2A Mutational Spectrum

Most reported pathogenic *CC2D2A* variants are coding-region truncating or missense variants [5,6]. Intronic splicing variants are increasingly recognized but remain underdiagnosed [6]. This case adds a functionally characterized non-canonical splice-site allele to the *CC2D2A* mutational spectrum and supports the assessment of selected NCSS variants in unresolved JS cases. The splicing defect observed here differs from the exon-skipping and canonical splice-site defects reported for other intronic *CC2D2A* variants [6], broadening the known range of disease-associated splicing mechanisms in this gene.

The second missense variant, c.4732G>A, is predicted to result in p.(Val1578Met), which lies in the C2 domain, where missense changes have been shown to impair ciliary integrity and signaling [23]. We did not experimentally validate the functional impact of p.(Val1578Met). On an independent allele basis, c.4732G>A remains within the VUS range because direct functional evidence is lacking. In this family, its trans configuration with the experimentally confirmed likely pathogenic c.439-5T>G variant and the concordant phenotypes in the proband and fetus are consistent with a compound heterozygous mechanism; however, these data do not establish the pathogenicity of the missense allele.

### 4.2. The Diagnostic Value of Functional Validation for NCSS Variants

NCSS variants are difficult to interpret in clinical practice [14,15]. Standard diagnostic pipelines focus on exonic and canonical splice-site variants, so NCSS variants are often missed or reported as VUS [6,8]. Although SpliceAI and MaxEntScan predicted an effect of c.439-5T>G on splicing, the prediction alone was not definitive, leading to its initial VUS classification. Functional assays provide direct evidence of splicing aberrations; consistent with the ClinGen SVI Splicing Subgroup recommendations, such RNA-level splicing evidence is captured through the PVS1 decision tree, whereas PS3 applies to independent downstream functional assays, facilitating variant reclassification [14,15,21]. Prospective implementation of RNA-based splicing analysis in clinical diagnostic laboratories has demonstrated that over half of VUS in splicing-related genes can be reclassified, most often upgraded as likely pathogenic or pathogenic [24].

In this case, the minigene assay, combined with patient RNA confirmation, provided direct evidence of aberrant splicing for a variant located outside the canonical ±1/±2 dinucleotides, with the local sequence supporting the creation of a cryptic AG acceptor at positions −6/−5. Following the recommendations of the ClinGen SVI Splicing Subgroup, such experimental data can be captured using the PVS1 decision tree, as the observed splicing defect is interpretable via the same loss-of-function framework applied to canonical splice-site variants [21]; the basis for retaining the Very Strong level for c.439-5T>G is detailed in Section 3.5. The data were therefore coded as PVS1 only; PS3 was not applied in addition, because both criteria would draw on the same experimental evidence.

### 4.3. Clinical Implications and Prenatal Diagnosis

In the largest *CC2D2A* cohort reported to date, normal intellectual efficiency was reported in 74% of assessed patients, and renal, retinal, and hepatic involvement was uncommon [5]. These figures are group-level observations: developmental delay was nearly constant in the same cohort, certain genotypes, such as homozygous p.Arg950*, were associated with a more severe phenotype [5], and the outcome of an individual patient cannot be predicted from such data. This variability is especially relevant in the prenatal setting, where group-level associations must not be taken to imply a favorable prognosis for an individual fetus. The proband in this study attends a regular school with learning support; her course is a single observation and should not be taken to predict the outcome of future pregnancies. Reclassification of c.439-5T>G as likely pathogenic was completed before the subsequent pregnancy and enabled prospective variant-directed prenatal testing, although c.4732G>A remained a VUS. Amniocentesis at 18 weeks showed that the fetus carried both variants. Because the missense allele remained a VUS, the molecular result alone was not unequivocally diagnostic; fetal brain MRI at 26 weeks was therefore incorporated into the assessment and showed cerebellar abnormalities consistent with JS. These findings informed reproductive counseling, and the trans configuration of the second allele supported, but did not establish, a biallelic molecular explanation.

### 4.4. Limitations

This study has several limitations. We validated c.439-5T>G splicing using a minigene assay in HEK293T cells and patient blood RNA but did not assess transcript stability in patient fibroblasts or evaluate nonsense-mediated mRNA decay (NMD), which may affect residual protein levels. This does not affect the PVS1 strength assignment, which under the PVS1 decision tree is based on the predicted position of the PTC relative to the NMD boundary rules rather than on experimental demonstration of decay [22]. We further note that this assessment was based on a single minigene transfection; low-level residual normal splicing below the detection limit of the assay cannot be formally excluded, and the absence of residual normal splicing is therefore attributed to the minigene system rather than to endogenous RNA. The pathogenicity of c.4732G>A (p.(Val1578Met)) rests on its trans configuration with the likely pathogenic c.439-5T>G variant (ACMG PM3); direct functional evidence for this missense change is lacking. Our findings derive from a single family. JS shows intrafamilial and interfamilial phenotypic variability even within the same gene [25], and additional cases are needed to determine the recurrence frequency of c.439-5T>G and its phenotypic range. Organ-specific assessment of the proband was incomplete: electroretinography, renal ultrasonography, and serum renal and liver function tests were not performed. Although the local sequence supports the creation of a cryptic AG acceptor at positions −6/−5, this mechanism has not been confirmed experimentally; targeted mutagenesis of the candidate acceptor dinucleotide or spliceosome assembly assays could provide direct evidence.

## 5. Conclusions

We showed that the non-canonical intronic variant *CC2D2A* c.439-5T>G causes aberrant splicing with 4 bp intron retention, resulting in a predicted frameshift and premature termination codon. This experimental evidence supported reclassification from VUS to likely pathogenic. The missense variant c.4732G>A (p.(Val1578Met)) remains a VUS on an independent allele basis; its trans configuration with the likely pathogenic allele supports, but does not establish, a biallelic molecular explanation in this family. These findings informed genetic counseling and variant-directed prenatal testing. This case illustrates the value of functional evaluation of selected NCSS variants when in silico predictions are inconclusive. For c.439-5T>G, the minigene assay, confirmed in patient RNA, defined the molecular consequence and supported reclassification under the ACMG/AMP framework. A similar approach may help clarify comparable splicing VUS in other families with JS or related ciliopathies.

## Figures and Tables

**Figure 1:**
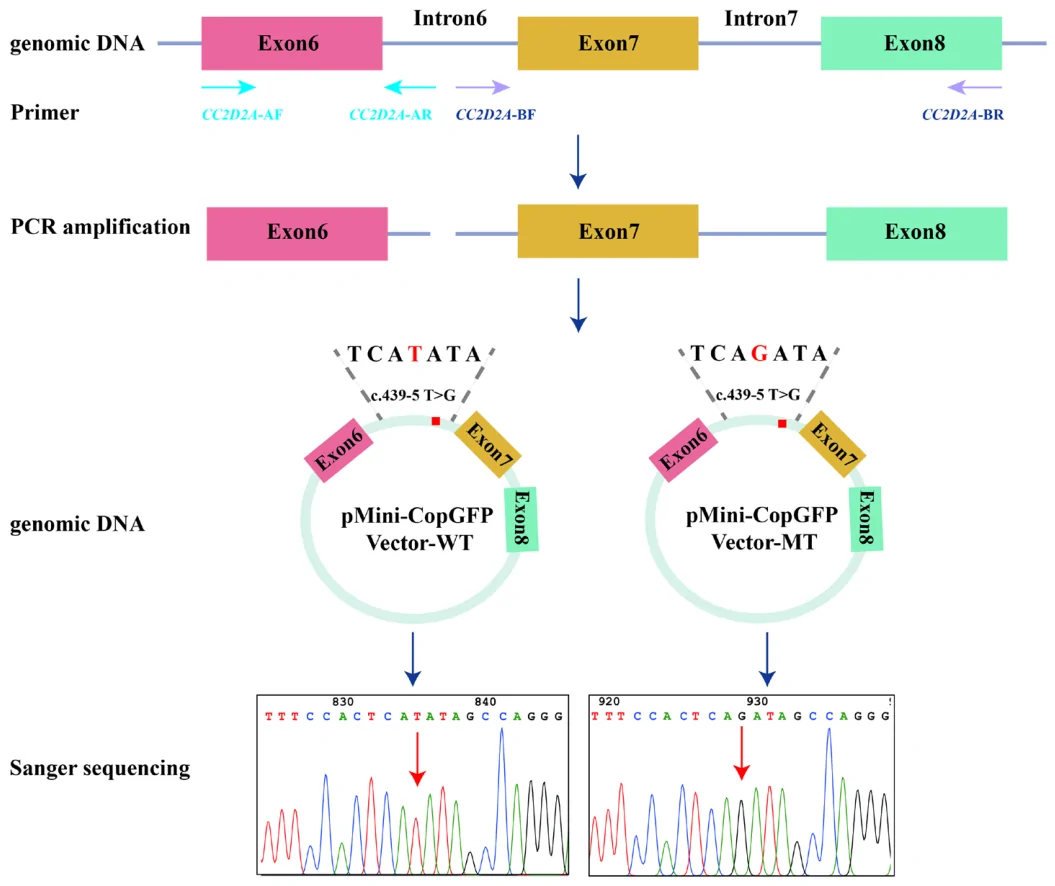
Schematic of minigene construction. Genomic fragments encompassing *CC2D2A* exons 6–8 were amplified from healthy control (WT) and proband (MT, c.439-5T>G) genomic DNA, then cloned into the pMini-CopGFP vector. The final constructs were confirmed by Sanger sequencing. Blue arrows indicate the cloning workflow and red arrows indicate the variant site.

**Figure 2:**
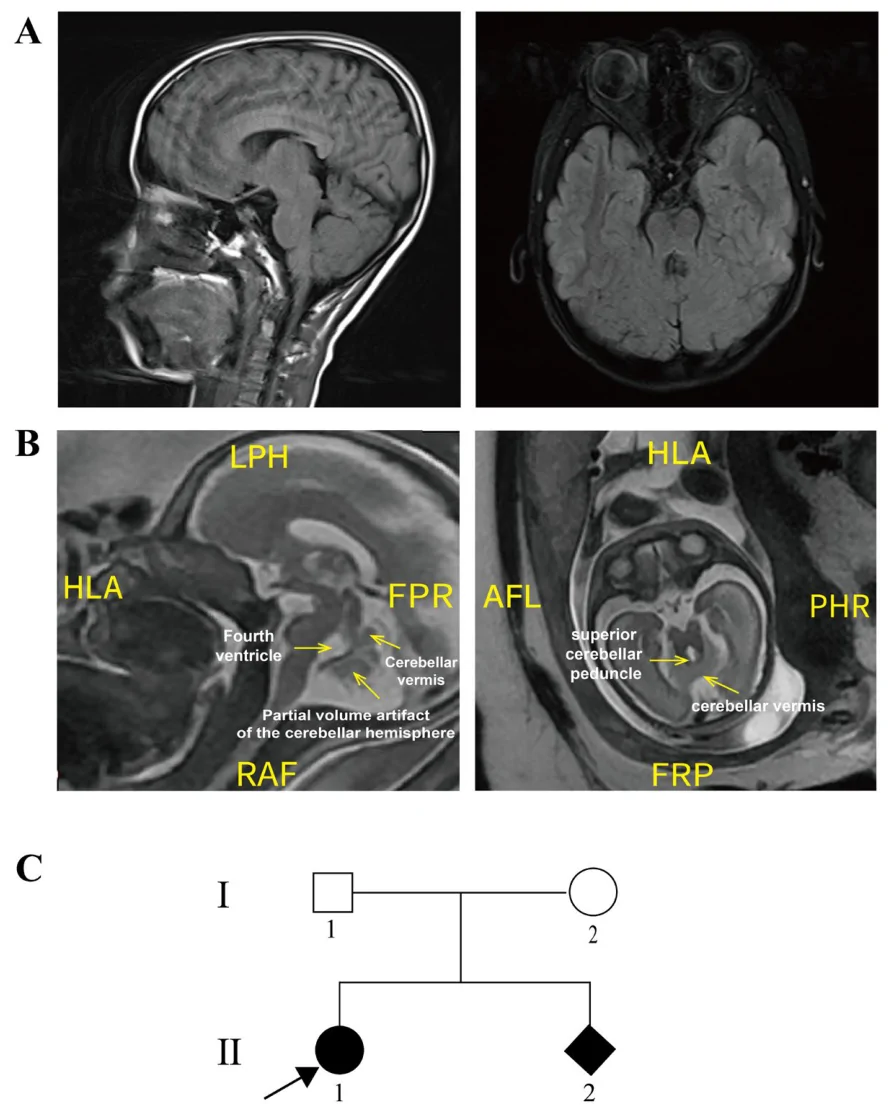
Clinical information of the proband and her family members. (**A**) Brain magnetic resonance imaging (MRI) of the proband. (**B**) Fetal brain MRI of the affected fetus at 26 weeks of gestation (SSFSE): axial image (right; slice thickness 3.0 mm) and sagittal image (left; slice thickness 3.6 mm). Labels indicate the thickened and elongated superior cerebellar peduncles, the small cerebellar vermis, and the abnormally configured fourth ventricle, consistent with a prenatal molar tooth sign. (**C**) Family pedigree. The proband (indicated by the arrow) and the affected fetus are both represented by filled symbols.

**Figure 3:**
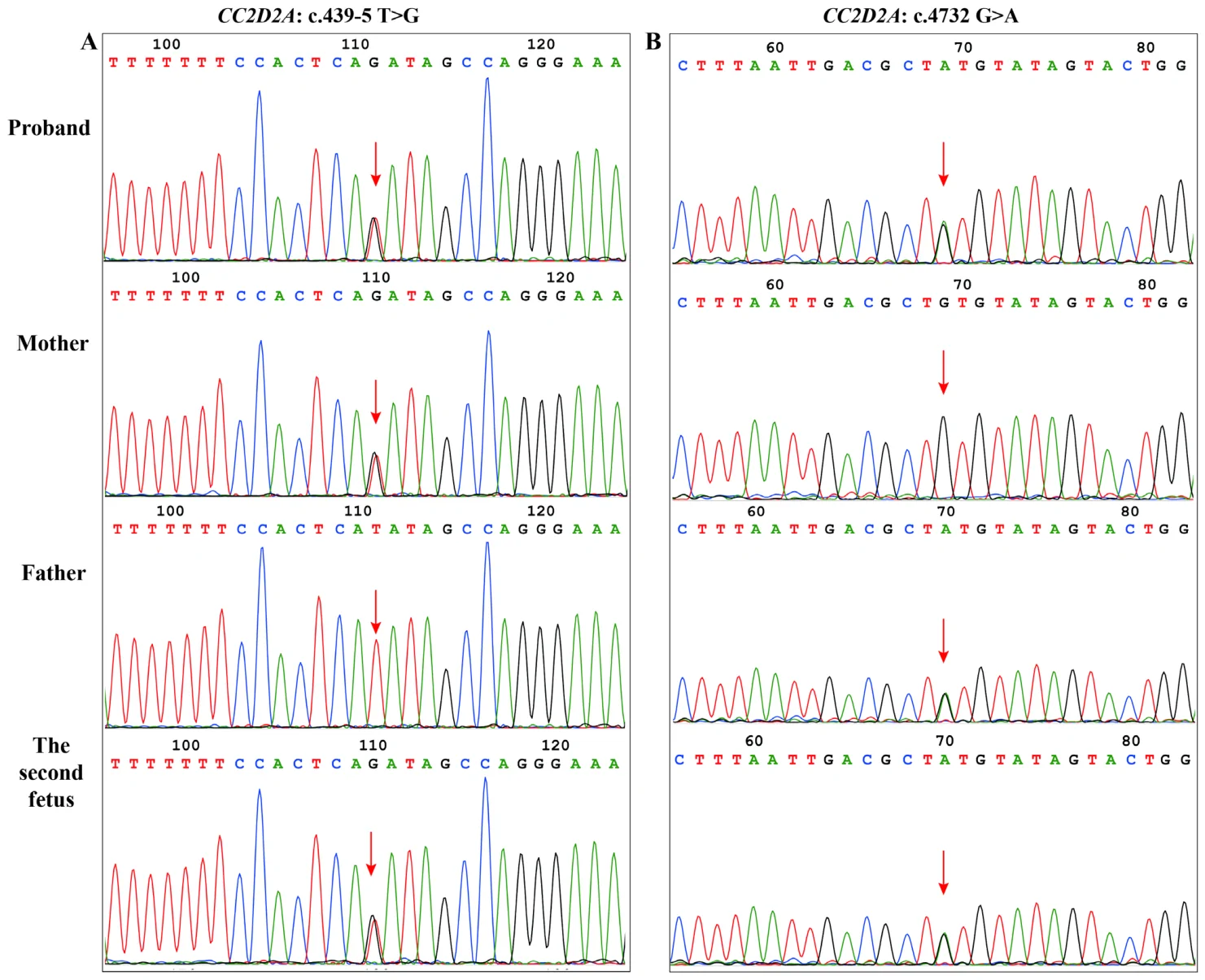
Sanger sequencing results of the proband and her family members. (**A**) The c.439-5T>G heterozygous variant was detected in the proband, the affected fetus, and their mother; the father carried the wild-type allele. (**B**) The c.4732G>A heterozygous variant was detected in the proband, the affected fetus, and their father; the mother carried the wild-type allele. Red arrows indicate the variant sites.

**Figure 4:**
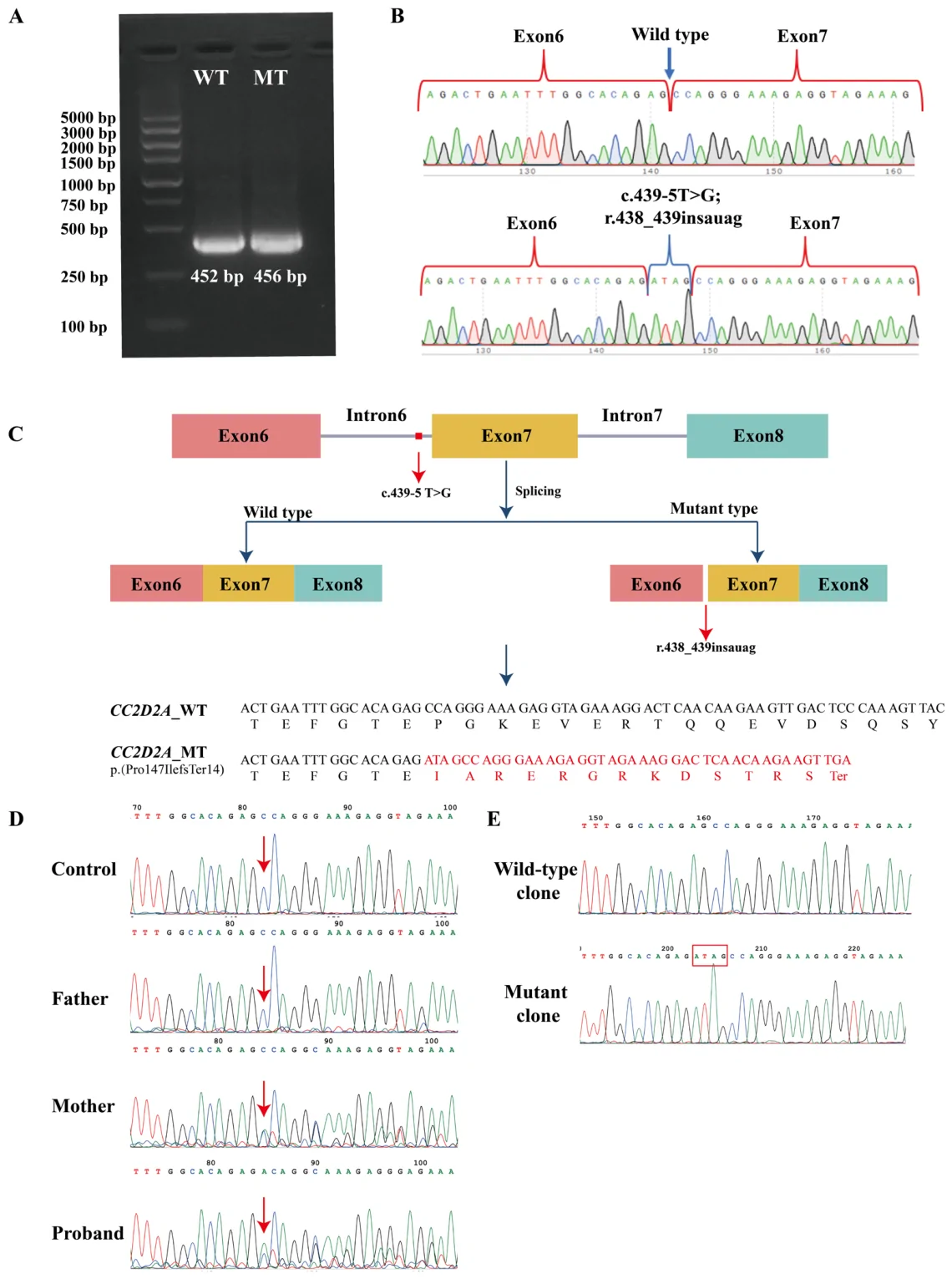
RT-PCR and minigene assays reveal aberrant splicing caused by c.439-5T>G. (**A**) RT-PCR of minigene-transfected HEK293T cells, showing single specific amplification products for the WT and MT constructs; the 4 bp difference between the 452 bp (WT) and 456 bp (MT) products is not resolvable on a 2% agarose gel and was established by Sanger sequencing. (**B**) Sequencing confirms retention of the intronic nucleotides c.439-4_439-1 (ATAG) from intron 6 in the MT construct (r.438_439insauag). (**C**) Predicted protein consequence: frameshift at codon 147 leading to premature termination (p.(Pro147IlefsTer14)). (**D**) Patient RNA validation by RT-PCR using the gene-specific primers *CC2D2A*-cDNA-F and *CC2D2A*-cDNA-R (Table 1); red arrows indicate the exon 6–7 junction. The control and father (wild-type) show clean junction sequences, whereas the proband and her mother (both heterozygous c.439-5T>G carriers) show overlapping peaks downstream of the junction, consistent with the coexistence of normal and aberrant transcripts. Red arrows indicate the variant sites. (**E**) TA cloning of the proband’s RT-PCR products: the wild-type clone shows normal exon 6–7 splicing, and the mutant clone shows the 4 bp ATAG retention (red box; c.439-4_439-1, r.438_439insauag).

**Table 1:** Primers for Sanger sequencing and RT-PCR.

Primer	Sequence	Application
439-F	GCAGCAGCAGTCAGAAGATAAG	Sanger sequencing
439-R	GTGCTCAATATATCTGCCTGTCT	
4732-F	TCCCTTCAGTTCTCTGGATTTC	
4732-R	GAATAGAGGCAGGAAGCTCATAA	
*CC2D2A*-AF	AAGCTTGGTACCGAGCTCGGATCCTCCAAAGCAGAAAGTGCATTGCTGCAGG	Recombinant plasmid construction
*CC2D2A*-AR	GAGATTGCAGGAGTAGCTGGGACTACAGGCACAAGC	
*CC2D2A*-BF	CCAGCTACTCCTGCAATCTCAGCTCACTGCAACCTC	
*CC2D2A*-BR	TTAAACGGGCCCTCTAGACTCGAGCATTTCCTTTCCTCCTCCTTGTGCAGGTG	
MiniRT-F	GGCTAACTAGAGAACCCACTGCTTA	RT-PCR of minigene
*CC2D2A*-RT-R	CATTTCCTTTCCTCCTCCTTGTGC	
*CC2D2A*-cDNA-F	AGCGAGGTCCAAAGCAGAAA	RT-PCR of patient endogenous RNA
*CC2D2A*-cDNA-R	CCCAATAGGAAGTCCTCGGC	

**Table 2:** ACMG/AMP evidence summary for *CC2D2A* c.439-5T>G.

Criterion	Strength	Points	Justification
PVS1	Very Strong	8	Minigene and patient RNA assays demonstrated aberrant splicing (r.438_439insauag) causing a frameshift, p.(Pro147IlefsTer14); the PTC lies in exon 7 of 37 on the MANE Select transcript and is predicted to trigger NMD; loss of function is the established mechanism of *CC2D2A*-related ciliopathy (ClinGen Definitive); no residual normal splicing was detected from the mutant minigene construct, in which the variant allele is expressed in isolation, within the assay’s detection limit
PM2	Supporting	1	Absent from population databases (ESP6500, ExAC, gnomAD)
Total		9	Likely pathogenic (6–9 points)

**Table 3:** ACMG/AMP evidence summary for *CC2D2A* c.4732G>A, p.(Val1578Met).

Criterion	Strength	Points	Justification
PM3	Moderate	+2	Confirmed in trans with the likely pathogenic c.439-5T>G variant in the affected proband, with phase verified by parental testing. PM3 was applied once; the fetus was treated as within-family segregation and was not counted as a separate observation
PM2	Supporting	+1	Absent from population databases (ESP6500, ExAC, gnomAD)
PP3	Supporting	+1	Missense prediction algorithms (SIFT, PolyPhen-2, MutationTaster, FATHMM) predict a deleterious effect at a conserved residue within a functional domain; scored once as a single correlated line of computational evidence
Total		4	VUS (0–5 points)

## Data Availability

The data presented in this study are available upon request from the corresponding author due to ethical and privacy restrictions related to patient data.
